# Should Pharmacies Be Included in Medication Reconciliation? A Report of Recurrent Valproic Acid Toxicity

**DOI:** 10.5811/cpcem.2016.12.33002

**Published:** 2017-03-15

**Authors:** B. Tate Cutshall, Samarth P. Shah, Megan A. Van Berkel, Shanise Patterson, L. Jeff Harris, Jessica V. Rivera

**Affiliations:** * Houston Methodist Hospital, Department of Pharmacy, Houston, Texas; † Methodist LeBonheur Healthcare-University Hospital, Department of Pharmacy, Memphis, Tennessee; ‡ Methodist LeBonheur Healthcare-South Hospital, Department of Pharmacy, Memphis, Tennessee; § University of Tennessee Health Science Center, Department of Emergency Medicine, Memphis, Tennessee

## Abstract

Including outpatient pharmacies in the medication reconciliation process upon hospital discharge is not commonly performed. This case highlights the consequences of a patient refilling a discontinued prescription for valproic acid (VPA). We present a 32-year old male found unresponsive after ingesting delayed release divalproex sodium. Cerebral edema was visualized on magnetic resonance imaging. Hemodialysis and levo-carnitine treatment led to improved mental status, and VPA was discontinued. The same patient presented with VPA overdose eight months later after he continued to fill an outdated prescription. This case highlights consequences of VPA toxicity; it also demonstrates an opportunity to improve patient safety and high-value care by collaborating with outpatient pharmacies in the medication reconciliation process upon hospital discharge.

## INTRODUCTION

Inaccurate medication reconciliation is the source of many medication-related misadventures leading to hospital admissions and patient morbidity and mortality.^1^ In randomized controlled studies, pharmacist-led discharge medication reconciliation interventions result in hospital cost avoidance and improve patient safety.^1^ Valproic acid (VPA) is an antiepileptic medication commonly used to treat seizures, bipolar disorder, and migraine headache.^2^ Its mechanism of action includes sodium channel inhibition, T-type calcium channel inhibition, suppression of glutamate, and inhibition of γ-aminobutyric acid (GABA) metabolism. VPA is available in a variety of dosage forms, and peak plasma concentrations are achieved rapidly (within 1–5 hours).^3^ With toxic ingestions, absorption and subsequent peak may be delayed; one case report reported peak serum levels 17 hours post ingestion.^4^ Therapeutic concentrations of VPA range from 50 to 100 μg/L, and it is 80–90% plasma protein bound.^3^ Elimination occurs via first-order kinetics with a half-life of 5–20 hours; however, this can be prolonged up to 30 hours with toxicity.^3^ At toxic levels, VPA can cause central nervous system (CNS) depression, respiratory depression, acute kidney injury, anion-gap metabolic acidosis, and electrolyte abnormalities (i.e. hypernatremia and hypocalcemia). VPA is also associated with hepatotoxicity, pancreatitis, hyperammonemic encephalopathy, cerebral edema, and blood dyscrasias such as leukopenia, anemia, and thrombocytopenia.

Treatment of VPA toxicity is largely supportive; however, it can include enhanced elimination methods such as charcoal hemoperfusion and hemodialysis (HD). HD is known to clear toxins that are water soluble, have low volume of distribution, and are not highly bound to plasma proteins. VPA exhibits high plasma protein binding with therapeutic concentrations; however, saturation of plasma proteins may occur in the setting of acute intoxication and result in increased free VPA concentrations amenable to HD.^5^–^6^ While published data are limited, high-flux HD has been shown to be sufficient without the need for concomitant charcoal hemoperfusion.^7^ Furthermore, HD should be considered to remove ammonia or correct severe metabolic disturbances during VPA toxicity.

Hyperammonemia caused by VPA toxicity is a complex process; it involves depletion of carnitine stores and ultimately results in inhibition of carbamoyl-phosphate synthetase, the primary enzyme responsible for ammonia incorporation into the urea cycle. The use of L-carnitine in the treatment of VPA-induced hyperammonemia is secondary to its ability to assist in the metabolism of long-chain fatty acids.

We present a patient with two presentations of VPA toxicity, eight months apart, each successfully treated with HD and L-carnitine. The cases presented provide insight on the detrimental effects that VPA toxicity can cause and review current evidence-based treatment options. Additionally, the second presentation sheds light on the unfortunate repercussions that an incomplete discharge reconciliation can have, namely the lack of patient care transition from inpatient to outpatient when a significant medication event occurred in hospital and subsequent medication changes were made.

## CASE REPORT

### First Presentation

A 32-year-old African-American male with a history of bipolar disorder, hypertension, and previous suicide attempts was brought to the emergency department (ED) with altered mental status (AMS). The patient ingested an unknown amount of his prescription medications with the timing unknown. He was prescribed lisinopril 10mg by mouth (PO) daily, hydrochlorothiazide 25mg PO daily, and divalproex sodium delayed release 500mg PO every morning and 1000mg PO nightly. Upon presentation, the patient was responsive only to painful stimuli with a Glasgow Coma Score (GCS) of 12. Vitals included a blood pressure of 152/70 mmHg and heart rate of 110 beats per minute. Computed tomography of the brain/head was unremarkable. However, magnetic resonance imaging (MRI) revealed cerebral edema and possible laminar necrosis.

On presentation, pertinent laboratory values included the following: VPA 481.9 μg/dL, lactate 6.9 mmol/L, ammonia 303 μmol/L, and platelets 135 × 10^3^ microL and serum creatinine 2.61 mg/dL. Other chemistries and liver function tests were within normal limits (WNL). The urine drug screen, serum alcohol level, acetaminophen level, and salicylate level were unremarkable.

The patient required intubation for AMS and acute respiratory failure, and a temporary dialysis catheter was emergently placed for HD. Prior to HD and three hours after initial presentation, VPA level rose to greater than 600 μg/mL (Figure).

The patient was dialyzed six hours after presentation for a total of six hours; post HD he was started on intravenous (IV) L-carnitine 1,300 mg q4h (15mg/kg/dose) based on literature recommendations and received lactulose 30 grams PO once.^8^ During his hospitalization, platelets reached a nadir of 63 × 10^3^ microL, but other pertinent laboratory results remained WNL. The patient improved clinically to include a GCS of 15, allowing successful extubation on hospital day 2. VPA was not to be continued upon discharge and he was transferred to a psychiatric facility for further evaluation.

### Second Presentation

During the second presentation, the patient was found unconscious and diaphoretic in his bedroom by his caregiver. He had AMS (GCS 12) and was unable to communicate effectively. Home medications included venlafaxine 75 mg PO daily, benztropine 2 mg PO daily and divalproex sodium DR 500 mg PO twice daily. Although he was not discharged on divalproex sodium during his last visit, he had continued to refill this prescription at his outpatient pharmacy. Notably, the patient also had been using marijuana regularly the previous week.

Baseline laboratory values on arrival include the following abnormalities: ammonia 48 μmol/L, VPA level 420 μg/mL, lactate 2.6 mmol/L, glucose 67 mg/dL, and platelets 127 × 10^3^ microL. Other laboratory values were WNL.

Although he did not immediately require HD, nephrology was emergently consulted in light of the complications of his previous admission. A repeat VPA level of 272 μg/mL was drawn five hours after the initial level before HD (Figure), and the patient was dialyzed for four hours. L-carnitine 2,640 mg PO q8h (32 mg/kg/dose) and lactulose 10 g PO four times a day were initiated. Laboratory findings one hour after HD included ammonia 84 μmol/L and VPA level 105 μg/mL. His mental status and symptoms improved (GCS 15), and the patient was able to follow commands appropriately. He was discharged on hospital day 2 to a psychiatric facility with instructions to continue L-carnitine 2,640 mg PO every eight hours given continued elevation in ammonia levels. This presentation scored a 10 on the Naranjo scale indicating a definite adverse drug reaction.^9^

## DISCUSSION

We present a unique patient with two separate presentations of VPA toxicity necessitating aggressive measures and treatment with HD and L-carnitine. On the first admission, cerebral edema was visualized on MRI and a peak VPA level of greater than 600 μg/mL was reduced to 199 μg/L at the end of a six-hour HD session. During the second admission, a peak level of 420 μg/mL decreased to 105 μg/mL after a four-hour HD session. While VPA is highly protein bound, plasma proteins become saturated during VPA toxicity, causing an increase in unbound VPA that contributes to the signs and symptoms of toxicity. These small molecules become amenable to elimination via HD allowing for more rapid decline in the serum concentration and subsequent improvement in symptoms of toxicity, as evidenced by the patient’s first presentation.^5^,^6^

Historically, charcoal hemoperfusion was used for the treatment of VPA toxicity.^9^ However, previous case reports describe the effectiveness of using HD alone. What remains unclear is the threshold in VPA concentrations where HD may be useful. Based on the literature available, the EXtracorporeal TReatments in Poisoning (EXTRIP) workgroup recommends dialysis in patients with a VPA concentration greater than 1,300 mg/L, the presence of cerebral edema, or shock.^7^ Dialysis may be used when VPA concentrations are greater than 900 μg/mL, in the presence of coma, respiratory depression requiring mechanical ventilation, acute hyperammonemia, or pH less than 7.1.^7^ Similarly, a review article evaluating extracorporeal elimination of VPA advises HD in severe VPA toxicity (coma or hemodynamic compromise) and a plasma VPA level >850 μg/mL.^9^ During our patient’s first presentation, the suggested criteria for HD were met due to the presence of cerebral edema on MRI and the need for mechanical ventilation. In the second presentation, AMS and his ingestion history drove the decision for HD. In a patient with therapeutic VPA concentrations, HD should not significantly impact VPA levels; it may be a viable option in acute toxicity by reducing free drug and improving clinical condition.

Hemodialysis is a viable option for treatment of VPA-induced hyperammonemia. VPA is metabolized primarily in the liver by means of glucuronic acid conjugation and oxidative pathways via the cytochrome P450 system. The major metabolites are 2-en-VPA, 4-en-VPA, and propionic acid derivatives which are active. 2-en-VPA has a long half-life and causes cerebral edema and coma, while 4-en-VPA causes reversible hepatotoxicity. Propionic acid is responsible for causing hyperammonemia by three proposed mechanisms. Its interaction with and depletion of carnitine impairs the transportation and metabolism of long-chain fatty acids. Also, it prevents glutamine production in the kidneys, which reduces ammonia levels in the brain. Lastly, it inhibits carbamoyl-phosphate synthetase, a hepatic mitochondrial enzyme responsible for eliminating ammonia within the urea cycle. The cumulative result is accumulation of ammonia, causing encephalopathy. L-carnitine has the ability to transport and metabolize long-chain fatty acids; thus, it has shown to be beneficial in VPA-induced hyperammonemia, especially in patients with hepatotoxicity, hyperammonemia, or significant CNS depression.^11^–^16^

An interesting aspect of our case was the reported increase in cannabis use during the week prior to the second VPA ingestion. To our knowledge, there are no reports of VPA toxicity caused by cannabis ingestion. However, cannabidiol, a component of marijuana, weakly inhibits the CYP2C9 pathway. In addition, delta-9-tetrahydrocannabinoid has high plasma lipoprotein binding. The potential for weak inhibition of VPA metabolism via CYP2C9 and displacement of protein binding due to cannabis could have contributed to the toxicity, but this interaction has not been studied.^17^

Of most interest, is the demonstration of the importance of involving a patient’s outpatient pharmacy when a medication is discontinued for toxicity. This patient’s VPA was discontinued upon discharge after the first overdose; unfortunately, measures were not put in place to prevent patient access to medication. Thus, he continued to fill this medication from his outpatient pharmacy. Including a pharmacist in hospital discharge medication reconciliation has been previously shown to decrease 30-day hospital readmission.^18^,^19^ This service is commonly provided to patients with multiple comorbidities and complicated medication regimens; however, a second occurrence of toxicity in this patient demonstrates that coordinating discharge care for patients with high-risk overdoses should be performed. Methods for reliably informing outpatient pharmacies of discharge medication reconciliation after acute care episodes are expected to improve patient safety.

## CONCLUSION

VPA toxicity causing hyperammonemic encephalopathy should be treated as a medical emergency. The use of HD is essential to decrease free VPA levels in some patients. Usage of L-carnitine at recommended doses of 50–100 mg/kg/day may aid in resolution of hyperammonemic encephalopathy due to its effect on the transportation and metabolism of long-chain fatty acids. While lactulose is a preferred treatment for hyperammonemia due to hepatic encephalopathy, routine use provides little benefit given the mechanism behind VPA-induced hyperammonemia. While this case presentation adds to the body of literature supporting high-flux HD for treatment of VPA toxicity, it further emphasizes the importance of including outpatient pharmacies upon medication reconciliation. This is especially relevant in cases where medications are discontinued due to toxicity or adverse effects. While we were able to manage the patient appropriately, proper communication after the first presentation could have prevented the second occurrence altogether. Having a multidisciplinary approach in improving communication between providers and outpatient pharmacies is vital in ensuring optimal patient care.

## Figures and Tables

**Figure:**
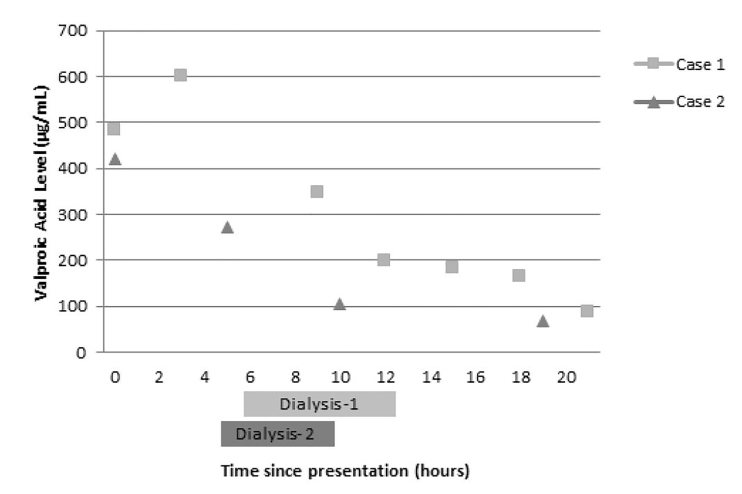
Serum valproic acid (VPA) levels during admission of patient with suspected VPA toxicity on two separate occasions
